# MCF-7 Human Breast Cancer Cells Form Differentiated Microtissues in Scaffold-Free Hydrogels

**DOI:** 10.1371/journal.pone.0135426

**Published:** 2015-08-12

**Authors:** Marguerite M. Vantangoli, Samantha J. Madnick, Susan M. Huse, Paula Weston, Kim Boekelheide

**Affiliations:** Department of Pathology and Laboratory Medicine, Brown University, Providence, Rhode Island, United States of America; Sanford Burnham Medical Research Institute, UNITED STATES

## Abstract

Three-dimensional (3D) cultures are increasing in use because of their ability to represent *in vivo* human physiology when compared to monolayer two-dimensional (2D) cultures. When grown in 3D using scaffold-free agarose hydrogels, MCF-7 human breast cancer cells self-organize to form directionally-oriented microtissues that contain a luminal space, reminiscent of the *in vivo* structure of the mammary gland. When compared to MCF-7 cells cultured in 2D monolayer culture, MCF-7 microtissues exhibit increased mRNA expression of luminal epithelial markers keratin 8 and keratin 19 and decreased expression of basal marker keratin 14 and the mesenchymal marker vimentin. These 3D MCF-7 microtissues remain responsive to estrogens, as demonstrated by induction of known estrogen target mRNAs following exposure to 17β-estradiol. Culture of MCF-7 cells in scaffold-free conditions allows for the formation of more differentiated, estrogen-responsive structures that are a more relevant system for evaluation of estrogenic compounds than traditional 2D models.

## Introduction

There is a large backlog of compounds for which adequate safety information is lacking, due largely to the time-intensive and expensive nature of animal-based toxicity testing [1]. Because of issues with reproducibility and predictability of animal models, there is a growing need to develop more differentiated and physiologically relevant *in vitro* test systems. *In vitro* systems have traditionally relied on cells cultured as a monolayer on plastic substrates, in stark contrast to the cell- and extracellular matrix-dense tissues *in vivo*. To address this gap, recent work has focused on the use of human cells and cell lines in physiologically relevant cell culture systems, including microfluidic on-chip models [2–4], 3-dimensional (3D) scaffolded, extracellular matrix-based models [5–8], and scaffold-free models [9, 10]. While on-chip models have been demonstrated to reconstruct *in vivo* biology in a desirable manner, they are labor intensive and difficult to adapt to high-throughput screening systems. Scaffolded models using laminin or collagen have been used for 3D cultures; however, several cell types have been shown to exhibit different phenotypes on each matrix [6, 11]. Overall, 3D cultures are of increasing importance, as they have been demonstrated to up-regulate tissue specific markers, regain tissue-specific functions and have different gene expression profiles when compared to cells cultured in traditional 2-dimensional (2D) systems [12–14].

Many studies have focused on the use of Matrigel and other basement membrane-rich matrices to culture human breast cells in 3D. Both normal and cancerous human breast cells have been grown in matrix-based culture models, with non-malignant MCF-10A cells forming mammary acini containing luminal spaces when cultured in Matrigel, and malignant MDA-MB-231 cells forming disorganized clusters of cells [15, 16]. While matrix-based culture models allow for the growth of cell lines in 3 dimensions, they have several limitations. First, previous work has demonstrated that growth of fibroblasts on a collagen-rich matrix leads to a different phenotype when compared to growth on a laminin-rich matrix [17], which makes the selection of a relevant matrix an extremely important part of study design and interpretation of results. Additionally, Matrigel is derived from Englebreth-Swarm mouse sarcomas [7], calling into question the ability of this system to recapitulate more “normal” environments, and Matrigel exhibits lot-to-lot variability that has the potential to introduce large irregularities in the cell culture system. Finally, when using matrix-based culture models, cells are generally seeded at low densities, which is different from the highly cellular nature of epithelial tissues *in vivo*.

In contrast to the matrix-based 3D culture models, there has been an increase in use of scaffold-free culture models. Scaffold-free culture models such as hanging drop and roller cultures allow cells to maximize cell-cell contacts, and much like matrix-based 3D cultures, cells grown in scaffold-free conditions generally up-regulate tissue-specific markers and become more differentiated than 2D cultures [10, 14, 18, 19]. However, it is difficult to control the size of aggregates formed using the roller culture method, calling into question the reproducibility of the method for uses including high-throughput screening. Additionally, models like the hanging drop, can be technically challenging to work with, as media changes and exposure to compounds can be difficult and time-consuming to perform, making these methods difficult to scale up for high-throughput experimentation [20].

Scaffold-free culture using non-adhesive agarose hydrogels allows cells to self-assemble and self-sort into 3D microtissues, free of the influence of extracellular matrix [9, 18, 21, 22]. Use of non-adhesive hydrogels allows for control of microtissue size, easy maintenance of the cultures, large numbers of microtissues per tissue plate, and the ability to easily culture multiple cell types in controlled ratios to form complex microtissues [9, 18]. Many cell types have been grown in these models, and the system produces large numbers of microtissues that are reproducible and maximize cell-cell contacts [9, 18].

A large majority of published *in vitro* studies focused on breast cancer and/or estrogen receptor biology have used the MCF-7 human breast cancer cell line [23–28]. MCF-7 cells are estrogen responsive, and are often used *in vitro* to study estrogen receptor positive breast cancers [29]. Despite their genomic instability, the sheer amount of existing literature makes MCF-7 cells a useful model to understand estrogen receptor and breast cancer biology. This study demonstrates that MCF-7 cells cultured in a 3D scaffold-free system using non-adhesive agarose hydrogels form microtissues that contain a luminal space. During culture in this system, MCF-7 cells up-regulate breast-specific markers when compared to traditional 2D culture systems. Additionally, 3D MCF-7 microtissues remain responsive to estrogen, an important benefit of using MCF-7 cells in this system. Furthermore, we find that the use of non-adhesive agarose hydrogels to culture breast epithelial cells results in a more differentiated, easy to manipulate cellular system, with potential application to studies in cell and cancer biology, and use as a screening platform.

## Materials and Methods

### Chemicals and Reagents

Cell culture media, supplements and SuperScript III First Strand Synthesis kit were purchased from Life Technologies, Inc (Grand Island, NY). Fetal bovine serum (FBS) was purchased from Atlanta Biologicals (Flowery Branch, GA) and dextran-coated-charcoal (DCC) stripped was purchased from Gemini Bioscience (Sacramento, CA). Estradiol (E2), dimethylsulfoxide (DMSO), Trizol and insulin were purchased from Sigma Aldrich (St. Louis, MO). Agarose was purchased from Fisher Scientific (Agawam, MA). Technovit 7100 was purchased from Heraeus Kulzer GmBH (Wehrheim, Germany).

### Cell Culture

MCF-7 cells (HTB-22) [30] were purchased from ATCC (Manassas, VA) and maintained in DMEM-F12 complete media supplemented with 10% fetal bovine serum (FBS), MEM nonessential amino acids, gentamicin and 10μg/mL insulin in a 5%CO_2_ incubator at 37°C. Media was changed every 2–3 days and cells were passaged when 65–80% confluent. MCF-7 cells were limited to use within the first 10 passages from the original purchased vial from ATCC, to control for genomic drift due to instability. As a comparison, MCF-7 cells purchased from the same lot from ATCC and cultured under the same media conditions were graciously provided by Dr. James Yager of Johns Hopkins University. For 2D cell samples, MCF-7 cells were plated at a density of 300,000 cells/well in 6-well plates and allowed to grow for 3 days. Cells were scraped into Trizol for RNA analysis and stored at -80°C. For imaging studies, all MCF-7 cells were grown on poly-L-lysine coated coverslips for 3 days at a seeding density of 300,000 cells/well in 6 well plates.

### 3D Scaffold-Free Cell Culture

Non-adhesive agarose hydrogels were made using molds (Microtissues Inc, Providence RI), equilibrated and seeded with cells as previously described [18]. MCF-7 cells grown in 2D flasks were trypsinized, counted and seeded into agarose hydrogels at a density of 600,000 cells/mL in complete growth media. Cells were allowed to settle into recesses for 30 minutes, with 2mL complete media added for growth. Media was changed every 2–3 days until collection.

### Estrogen Treatment of MCF-7 Cultures

For 2D samples, MCF-7 cells were seeded in 6-well plates at a density of 300,00 cells/well and allowed to grow for 3 days in 10% complete media. Cells were then placed in phenol-red free DMEM-F12 treatment media supplemented with 5% dextran-charcoal stripped fetal bovine serum, MEM nonessential amino acids, gentamicin and 6ng/mL bovine insulin for 48 hours. MCF-7 cells were seeded into agarose hydrogels and grown for 7 days in 10% complete media, as described previously, with media changes every 2–3 days. Following 7 days of growth, gels were washed twice with PBS and transferred to fresh treatment media for 48 hours. Following 48 hours, MCF-7 2D and 3D microtissues were exposed to estradiol or vehicle control (dimethylsulfoxide) for 8 hours, and collected in Trizol for gene expression analysis.

### Preparation of Frozen Sections

Microtissues within agarose hydrogels were fixed in 10% formalin overnight at +4°C. For autophagy staining, hydrogels were fixed in ice-cold methanol for 20 minutes. Following fixation and 2 washes with phosphate-buffered saline (PBS), samples were placed in a 15% sucrose/PBS solution for at least 3 hours at room temperature and then moved to a 30% sucrose/PBS solution overnight at +4°C. Samples were then placed in optimal cutting temperature compound (Fisher Scientific, Tissue–Tek, No. 14-373-65) and frozen on dry ice before sectioning. Embedded samples were stored at -80°C until use. Samples were sectioned at 8μm, melted to the slide and refrozen to affix to the slide. Slides were stored at -80°C until immunostaining.

### Immunofluorescent Staining of Frozen Sections

Frozen sections affixed to slides were allowed to warm to room temperature and then fixed for 5 minutes in 4% paraformaldehyde. Slides were washed with PBS three times (5 minutes each) at room temperature, and then permeabilized with 0.25% Triton X-100 for 10 minutes at room temperature. Samples were washed in PBS as before, and blocked with 1% goat serum (Sigma, No. G9023), 3% bovine serum albumin (Sigma, No. A7906) and 0.3 M glycine for 1 hour at room temperature. Slides were incubated overnight at +4°C with primary antibody at the dilutions summarized in S1 Table. Samples were washed with PBS and incubated with secondary antibody (S1 Table) for 1 hour. For F-actin staining to visualize the cytoskeleton, sections were incubated with rhodamine phalloidin (Invitrogen, R415, 1:500 dilution) for 25 minutes. Slides were washed as above and stained with Hoechst 33342 (Molecular Probes, No. H1399, 1:1000 dilution) for 30 minutes at room temperature. Coverslips were mounted using Prolong Gold Antifade reagent (Life Technologies, P36934) and stored until imaging in the dark at +4°C.

### Immunofluorescent Staining of Monolayer Cultures

MCF-7 cells were seeded at low density and grown for 3 days in 10% complete media on poly-l-lysine coated coverslips. Coverslips were fixed in 4% paraformaldehyde for 10 minutes at room temperature and then washed twice with 1x PBS. Following washes, samples were permeabilized for 15 minutes in 0.25% Triton X-100, then washed with PBS. Samples were blocked with 1% goat serum (Sigma, No. G9023), 3% bovine serum albumin (Sigma, No. A7906) and 0.3 M glycine for 1 hour at room temperature. Slides were incubated overnight at +4°C with primary antibody at the dilutions summarized in S1 Table. Samples were washed with PBS and incubated with secondary antibody (S1 Table) for 1 hour. For counterstaining, coverslips were incubated with rhodamine phalloidin and Hoechst 33342 as above. Coverslips were mounted as described above.

### TUNEL Staining of Frozen Sections

Formalydehyde-fixed frozen sections were allowed to warm to room temperature, washed with PBS three times and then permeabilized for 10 minutes using 0.25.% Triton X-100. Sections were then stained using the Click-iT TUNEL Alexa Flour 647 Imaging Assay Kit (Life Technologies, C10247) and compared to DNAse-treated positive controls. Coverslips were mounted using Prolong Gold Antifade reagent (Life Technologies, P36934) and stored until imaging in the dark at +4°C.

### Staining and Imaging of 3D Intact Cultures

Samples were fixed *in situ* overnight in 4% paraformaldehyde (PFA) at 4°C. Following fixation, samples were washed 3 times in 1X PBS, permeabilized in 0.25% Triton X-100 for 15 minutes while rocking and washed again with PBS. Samples were incubated with rhodamine phalloidin (1:500 dilution, Invitrogen) for 30 minutes, stained with DAPI (1:1000, Invitrogen) for 15 minutes and stored in the dark at 4°C until imaging on an Olympus FV1000-MPE multiphoton microscope.

### Histological Examination

For cells grown in 2D, cells on coverslips were fixed in 4% PFA for 10 minutes and stained with periodic acid-Schiff’s reagent followed by hematoxylin counter stain (PASH). For 3D microtissues, samples were fixed in 4% PFA overnight at 4°C, and then embedded in glycol methacrylate (Technovit 7100; Heraeus Kulzer GmBH, Wehrheim, Germany) for histological examination. Sections were cut at a thickness of 3μm, affixed to slides and stained with PASH. Samples were viewed and images taken on an Aperio ScanScope CS (Aperio Technologies, Vista, CA) at ×40 magnification.

### Transmission Electron Microscopy

Microtissues were fixed for 2 hours at room temperature in 2% gluaraldehyde in 0.1 M sodium cacodylate buffer, with 0.1 M sucrose, pH 7.4. Samples were then rinsed with buffer and post fixed with 1% osmium tetroxide in buffer for 30 minutes at room temperature. Samples were dehydrated through a series of graded ethyl alcohols for 15 minutes each, with three changes of 100% ethanol for 10 minutes each. The samples were then infiltrated with 1 part Spurr embedding medium (Electron Microscopy Science, Hatfield, PA), and 2 parts ethanol for 3 hours, overnight with a 1:1 Spurr to ethanol, solution, placed in a 3:1 Spurr to ethanol solution for 3 hours and then 100% Spurr for 6–8 hours. Samples were then embedded in molds and polymerized at 60°C. Blocks were sectioned at a thickness of 85 nm on a Reichert Ultramicrotome with diamond knife, placed on copper grids, and stained with uranyl acetate and lead citrate. Sections were viewed with a Phillips 410 transmission electron microscope equipped with an Advantage HR CCD camera. Images were acquired with Advanced Microscopy Techniques imaging software.

### Western Blot Analysis

Spheroids were prepared as previously described after 7 days of growth, and MCF-7 cells grown in 2D were scraped into PBS. Samples were collected, centrifuged and washed twice with ice-cold PBS. Samples were then lysed using RIPA buffer with HALT protease and phosphatase inhibitor (Thermo Scientific). Protein concentrations were measured using the DC Protein Assay (BioRad) per manufacturer’s instructions. Samples were prepared and 20 μg loaded into SDS-PAGE gels. Following gel electrophoresis, a transfer was performed using PVDF membranes (Thermo Scientific). Membranes were blocked and probed with antibodies as described in S2 Table. Membranes were developed using ECL (Thermo Scientific). The results of 3 individual experiments were quantified using densitometry in ImageJ, as previously described [31].

### RNA Isolation, RT-PCR and PCR Array Analysis

Microtissues were collected from hydrogels by centrifugation, pelleted and lysed in Trizol. Samples were then isolated using the RNEasy Mini Kit (Qiagen) per manufacturer’s instructions. For use in quantitative real-time PCR (qRT-PCR), cDNA was made using the SuperScript III First Strand Synthesis system per manufacturer’s instructions. qRT-PCR was performed to determine expression levels of progesterone receptor (PgR, forward primer (5’ to 3’): ACCCGCCCTATCTCAACTACC, reverse primer: AGGACACCATAATGACAGCCT), growth response to estrogen in breast cancer (GREB, forward primer: AATGGGTCCGGCTGTTTTC, reverse primer: CCAGTTGTTGGCACTTCGG) and normalized to ribosomal protein, large P0 (RPLP0, forward primer: GTGTTCGACAATGGCAGCAT, reverse primer: GACACCCTCCAGGAAGCGA). The delta-delta-CT method was used to determine changes in mRNA expression compared to untreated control. Data was plotted in GraphPad Prism software and two-way ANOVA used to determine statistical significance between 2D and 3D samples.

A custom SABiosciences PCR Array (Qiagen) was created to include a list of 84 genes generated from literature to evaluate estrogen receptor signaling, breast and ductal morphogenesis, cellular growth and differentiation, proliferation, tumor progression and epithelial to mesenchymal transition as well as 5 housekeeping genes and contamination controls (S3 Table). Samples were prepared per manufacturer’s instructions, and were added to 384-well plates using an epMotion 5075 automated pipettor (Eppendorf). Plates were run on an ABI 7900HT machine using cycling conditions recommended by the manufacturer.

### Statistical Analysis

The raw PCR cycle (Ct) values were imported into the R statistical environment [32]. Raw PCR cycles were normalized (dCt) using the SLqPCR package [33] to optimize selection of house-keeping genes. To compare the expression of 2D cell cultures (day 3) against 3D culture cells (day 7), we used the limma package in R [34] to construct a linear model of the adjusted dCt values. Specifically we used the empirical Bayes statistic (eBayes) of the linear model (lmfit) and then selected the genes with statistically significant changes between cultures as determined by an adjusted significance p-values for multiple experiments (q-value), q < = 0.05 [35].

## Results

**Fig 1:**
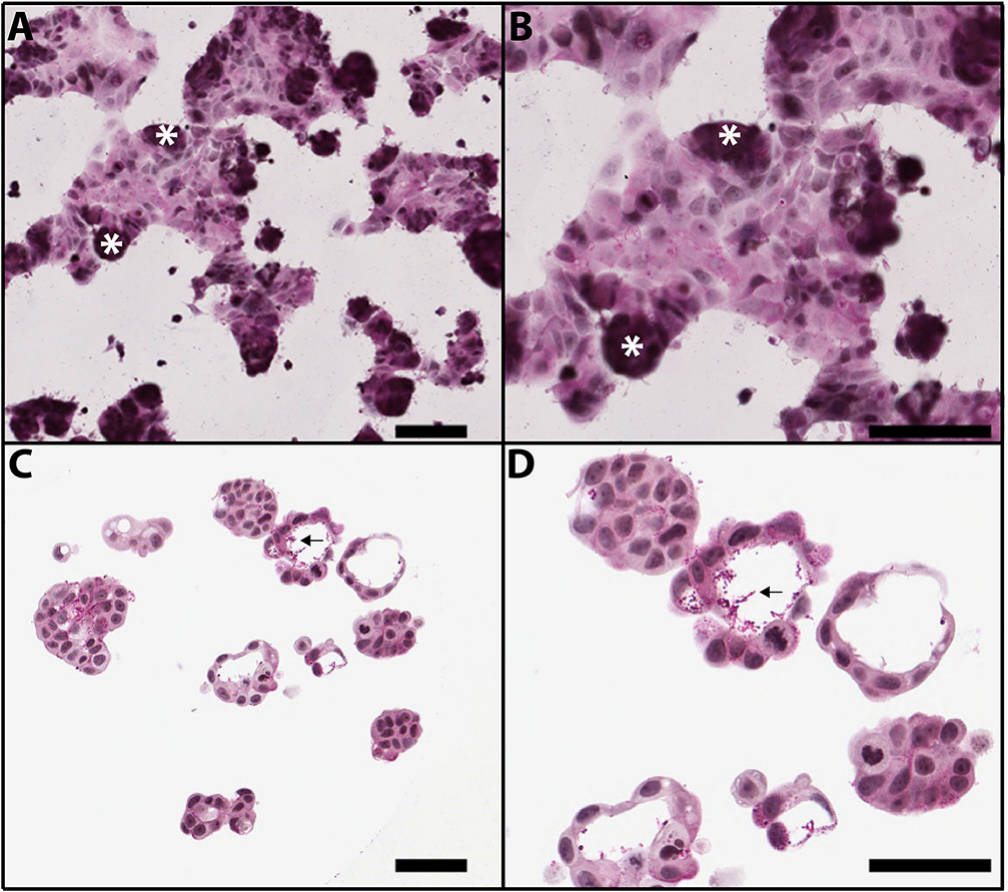
Morphology of MCF-7 cells grown in 2D and 3D cultures. MCF-7 human breast cancer cells grown in 2D monolayer culture for 3 days on poly-L-lysine coated coverslips and stained with periodic acid-Schiffs and hematoxylin (PASH) under low (A) and high (B) magnification. Cells exhibit typical cobblestone morphology, with several aggregates of cells (asterisks). MCF-7 human breast cancer cells grown in scaffold free non-adhesive agarose hydrogels for 7 days form microtissues with luminal spaces containing PAS-positive secretions (arrows) under low (C) and high (D) magnification. Scale bar = 50μm.

When grown on coated coverslips, MCF-7 cells exhibited typical 2D morphology, growing in mostly flat sheets with some piles of cells (Fig 1A and 1B). In contrast, MCF-7 cells sourced from Brown University and grown in non-adhesive agarose hydrogels formed 3D microtissues with multiple cellular aggregates per well containing luminal spaces following 7 days of culture (Fig 1C and 1D). MCF-7 microtissues possessed luminal spaces surrounded by a cell layer 1–2 cells thick (Fig 1D). Following 7 days in culture, the luminal spaces contained periodic acid-Schiff’s (PAS) positive secretions, indicating that carbohydrate-positive materials were being secreted into the lumens (Fig 1D). MCF-7 cells attained from Dr. James Yager were grown in 3D agarose hydrogels in identical culture conditions formed microtissues with cord-like structures and few luminal spaces following 5, 7, and 10 days of growth (S1 Fig). Due to the luminal structure, Brown University MCF-7 cells were selected for further characterization.

**Fig 2:**
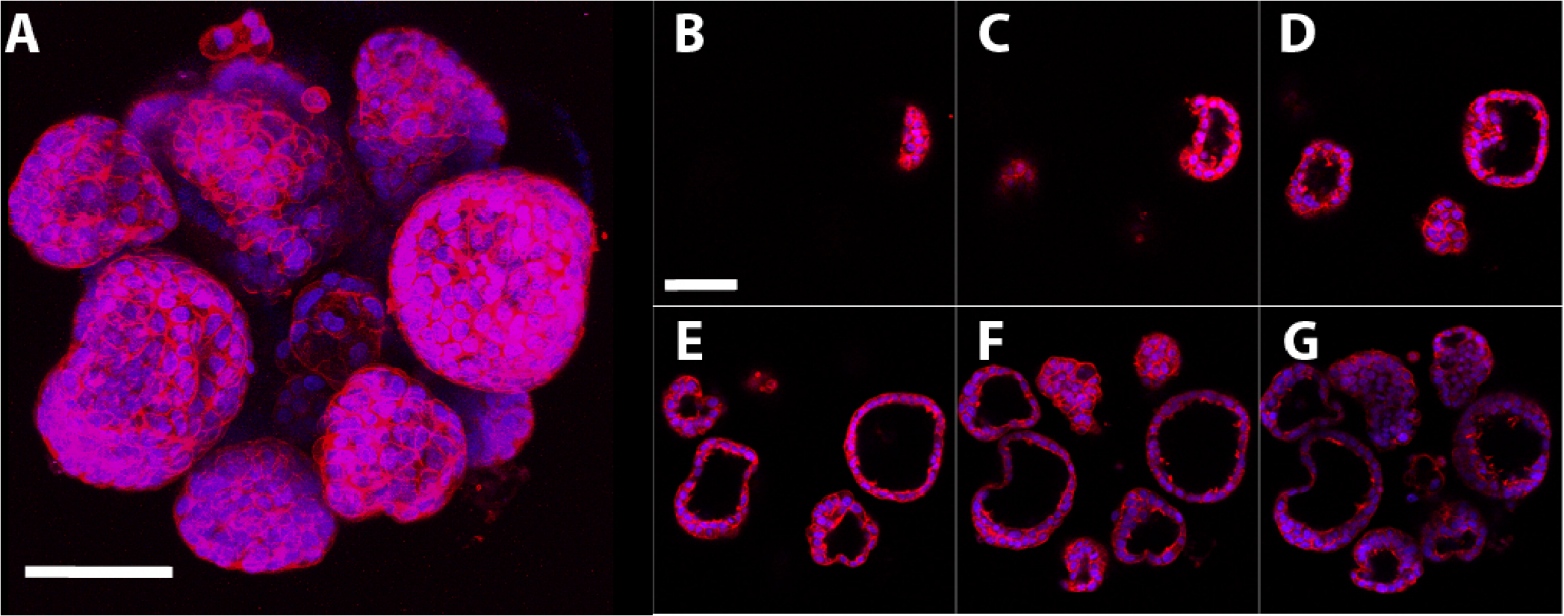
3D structure of 7-day MCF-7 microtissues. Intact MCF-7 microtissues were stained with rhodamine phalloidin (red; F-actin) and DAPI (blue; nuclear stain) and imaged on a confocal microscope, with steps taken in the Z direction every 5μm. Individual images were compiled to make a maximal projection image, demonstrating the morphology of the microtissues (A). Sequential images at intervals of 15μm in the Z direction demonstrate that microtissues contain luminal spaces (B-0μm, C-15μm, D-30μm, E-45μm, F-60μm, G-75μm). Scale bar = 80μm.

Imaging of the intact microtissues stained with rhodamine phalloidin (actin cytoskeleton) and DAPI (nuclear) stains revealed that after 7 days in culture, MCF-7 cells aggregated and organized into microtissues (Fig 2A). MCF-7 cells self-assembled into multiple microtissues per well that were 70–150μm in diameter, and these microtissues contained luminal spaces (Fig 2B–2G).

**Fig 3:**
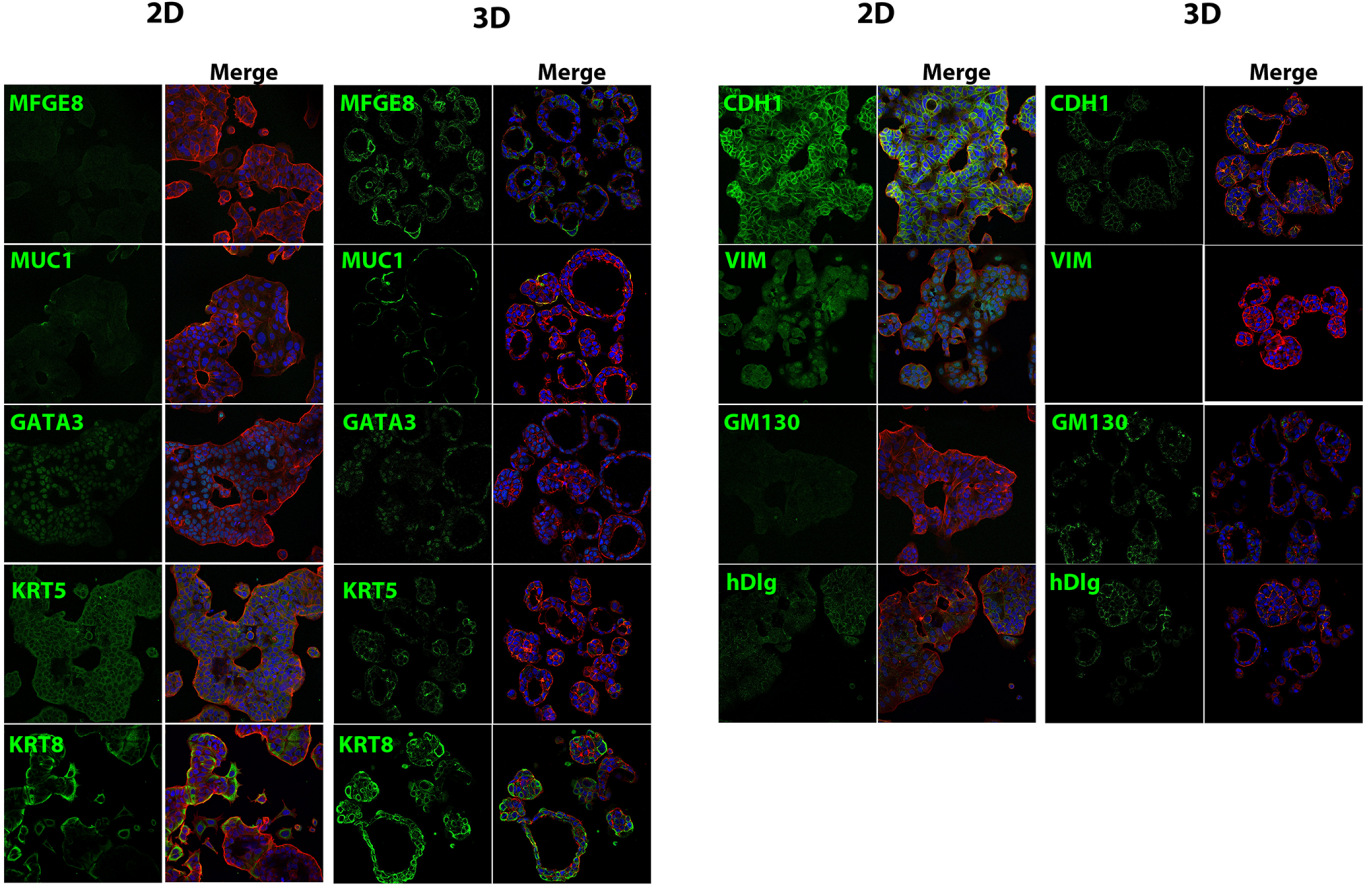
Expression of luminal and epithelial markers in 3D MCF-7 microtissues. Three day monolayer cultures and cryosections of MCF-7 cells grown for 7 days in agarose hydrogels were stained for epithelial and luminal markers (green), with f-actin rhodamine phalloidin (red) and Hoechst 33342 nuclear stain (blue) counterstains. Monolayer cultures exhibited diffuse staining for milk fat globule EGF8 (MFGE8) while 3D cultures showed intense, localized staining at the basal surface. Mucin 1 (MUC1) staining was faint and localized to select cells in 2D cultures with more intense basal staining in 3D microtissues. Expression of the luminal transcription factor trans-acting T-cell-specific transcription factor (GATA-3) was localized to the nucleus in both 2D and 3D cultures. Keratin 5 (KRT5) staining was diffuse in 2D cultures and more concentrated in specific cells in 3D cultures. There was high expression of keratin 8 (KRT8) in monolayer cultures, and throughout the microtissues (S, T). MCF-7 cells cultured in 2D demonstrated diffuse staining throughout the cytoplasm for e-cadherin (CDH1), while cells grown in agarose hydrogels displayed localized at cell-cell contacts. Mesenchymal marker vimentin (VIM) was present in the cytoplasm and nucleus of monolayer cultures and not detectable in 3D cultures. The Golgi marker GM130 displayed diffuse staining in monolayer culture but was punctate in MCF-7 microtissues, with localization at the apical surface in select cells, and in other was basolaterally oriented. Human disc large (hDlg) displayed diffuse staining in 2D cultures, but was expressed at cell-cell contacts and the basal surface in 3D cultures.

MCF-7 cells grown for 3 days as monolayers or 7 days in 3D scaffold-free culture were stained for markers of breast and epithelial cell differentiation (Fig 3). Monolayer cultures of MCF-7 cells displayed diffuse staining for milk fat globule-EGF factor 8 protein (MFGE8), while 3D cultures showed strong staining along the outside of the microtissues, with select cells staining more strongly. Mucin 1 (MUC1) was localized to the cell membrane of several cells in 2D cultures, while 3D cultures exhibited similar exterior localization of MUC1. Expression of trans-acting T-cell-specific transcription factor (GATA-3) was localized to the nuclei in both 2D and 3D cultures. The basal cell marker keratin 5 (KRT5) was diffusely distributed in MCF-7 monolayers, while in 3D cultures staining was localized close to the cytoskeleton of select cells. In 2D cultures, MCF-7 cells exhibited staining for the luminal epithelial marker keratin 8 (KRT8) at the leading cell edge, while there was high expression of KRT8 throughout MCF-7 microtissues. MCF-7 cells cultured in 2D displayed intense cytoplasmic staining for the epithelial marker E-cadherin (CDH1) while staining was localized to cell-cell contacts in 3D cultures. The mesenchymal marker vimentin (VIM) was present in the nuclear and cytoplasmic compartments of 2D MCF-7 cultures and was not present in 3D cultures. Golgi protein (GM130) showed diffuse staining throughout 2D cultures with small areas of punctate staining, while 3D cultures exhibited punctate staining with varying orientation, with many cells having staining present on the exterior surface, and others with staining on the apical and lateral sides. Human disc large (hDlg) staining in 2D cultures was mostly localized at cell-cell contacts, but cells did display cytoplasmic staining. 3D cultures stained for hDlg at cell-cell contacts as well as the basolateral surface.

RT-PCR analysis using a custom PCR array (S4 Table) was performed to determine differences in gene expression between MCF-7 cells cultured for 3 days in 2D monolayer culture and MCF-7 cells cultured in 3D for 7 days in agarose hydrogels (Table 1 and S4 Table). Gene expression analysis revealed statistically significant up-regulation of keratin 8 (KRT8, q = 0.036), keratin 19 (KRT19, q = 0.013), snail family zinc finger 1 (SNAI1, q = 0.023), milk fat globule-EGF factor 8 protein (MFGE8, q = 0.033), g-protein coupler estrogen receptor (GPER, q = 0.036), leucine rich repeat containing 15 (LRRC15, q = 0.036), mucin 1 (MUC1, q = 0.039) and WNT1 inducible signaling pathway protein 2 (WISP2, q = 0.013) in 3D MCF-7 microtissues compared to cells cultured in monolayer (Table 1). Additionally, mRNA expression of bone morphogenic protein 7 (BMP7, q = 0.0019) cystatin E/M (CST6, q = 0.0065), amphiregulin (AREG, q = 0.018_, apolipoprotein D (APOD, q = 0.023), estrogen receptor beta (ESR2, q = 0.025) and keratin 14 (KRT14, q) was significantly decreased in 3D cultures when compared to 2D cultures (Table 1). Expression of cytochrome P450 19A1 (CYP19A1, q = 0.033), vimentin (VIM, q = 0.33), FBJ murine osteosarcoma viral oncogene homolog (FOS, q = 0.033) and cyclin D2 (CCND2, q = 0.036) was decreased in 7 day 3D cultures compared to cells grown in 2D monolayer (Table 1). Complete PCR results are summarized in S4 Table.

**Table 1:** Gene expression changes in 3D cultures compared to 2D cultures.

Full Name	Abbreviation	q-value	fold change
Snail family zinc finger 1	SNAI1	0.02339	47.18
Keratin 19	KRT19	0.01305	3.56
Wnt-induced signaling protein 2	WISP2	0.01305	3.50
Leucine-rich repeat containing 15	LRRC15	0.03640	2.91
Milk fat globule-EGF factor 8	MFGE8	0.03253	2.56
G-protein coupled estrogen receptor	GPER	0.03613	2.48
Keratin 8	KRT8	0.03613	2.29
Mucin 1	MUC1	0.03950	2.21
Cyclin D2	CCND2	0.03613	-2.31
Vimentin	VIM	0.03253	-2.56
FBJ murine osteosarcoma viral oncogene homolog	FOS	0.03253	-2.57
Amphiregulin	AREG	0.01822	-3.20
Apolipoprotein D	APOD	0.02258	-3.98
Estrogen receptor alpha beta	ESR2	0.02545	-4.23
Cytochrome P450 19A1	CYP19A1	0.03269	-4.27
Cystatin E/M	CST6	0.00649	-4.35
Bone morphogenic protein 7	BMP7	0.00190	-6.94
Keratin 14	KRT14	0.03613	-29.19

**Fig 4:**
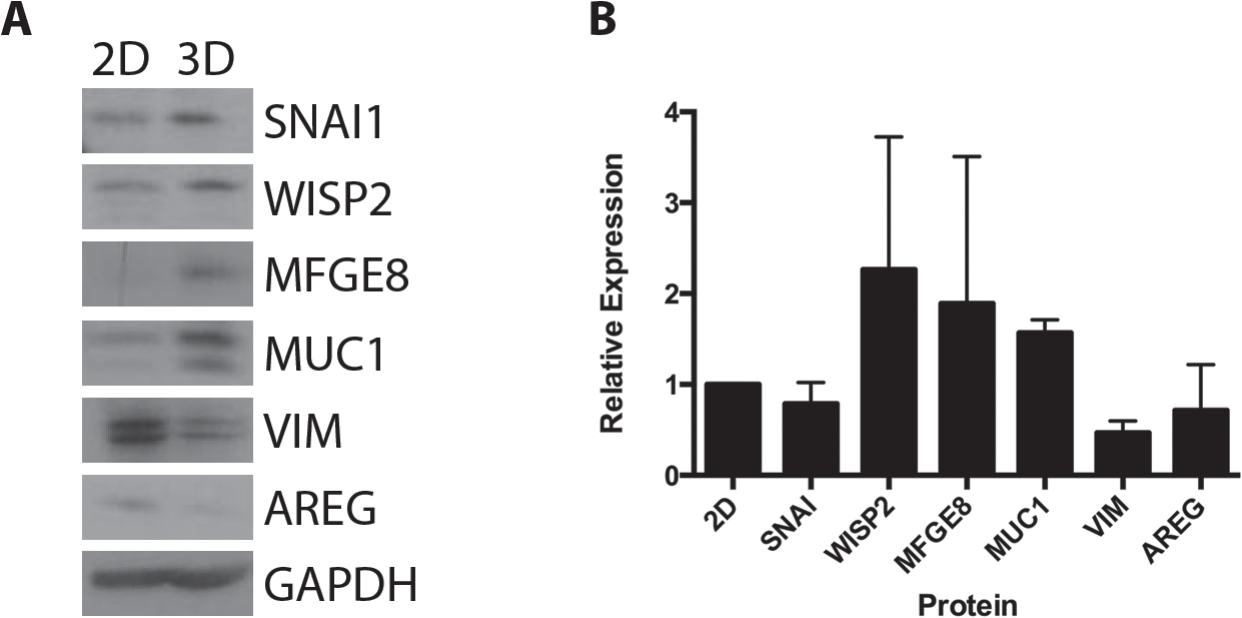
Protein expression of significantly increased transcripts in MCF-7 cells grown in 2D and 3D. MCF-7 cells were grown for 3 days in 2D culture or for 7 days in 3D culture. In agreement with increased mRNA expression, MCF-7 cells have increased protein expression of SNAI1, WISP2, MFGE8 and MUC1 and have decreased expression of VIM and AREG (A). Results from three individual experiments were quantified using ImageJ and analyzed using a one-way ANOVA (B), and are represented as relative expression compared to 2D cultures.

To verify the results of RT-PCR, protein expression was examined using Western blotting (Fig 4A). 3D cultures had increased expression of SNAI1, WISP2, MFGE8 and MUC1 when compared to MCF-7 cells cultured in 2D. When compared to 2D cultures, 7 days MCF-7 microtissues had decreased protein levels of VIM and AREG. Changes in expression were not significant across 3 individual experiments (Fig 4B), though trends were apparent.

**Fig 5:**
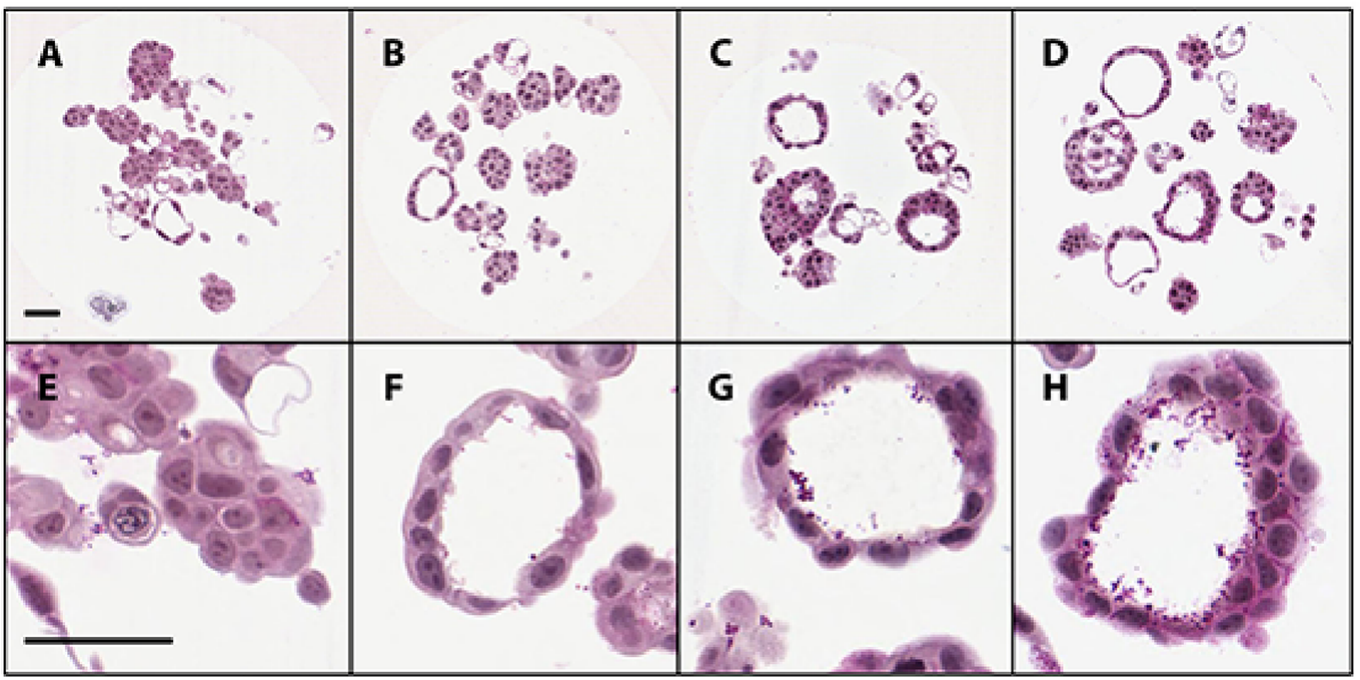
Morphology of MCF-7 microtissues over time in culture. MCF-7 cells aggregate into small groups forming microtissues following one day of growth (A, E) and have luminal spaces as early as 3 days in culture (B, F). Following 5 days in culture, luminal spaces are well developed and contain PAS-positive secretions (C, G). PAS-positive secretions continue to collect within the luminal spaces at 7 days (D, H). There is a lack of apoptotic debris in the luminal spaces of microtissues during this period. Scale bar = 50μm.

To determine the timing of lumen formation in MCF-7 microtissues, samples were embedded, sectioned and stained with PASH after 1, 3, 5, and 7 days of growth in non-adhesive agarose hydrogels. MCF-7 microtissues aggregated into small groups after 24 hours in culture (Fig 5A and 5E) and these microtissues developed luminal spaces by 3 days in culture (Fig 5B and 5F). After 5 days in culture, luminal spaces were well developed, and began to accumulate PAS-positive secretions in the luminal spaces (Fig 5C and 5G), which were increased after 7 days (Fig 5D and 5H). There was no apoptotic or necrotic material in the luminal spaces of MCF-7 microtissues.

**Fig 6:**
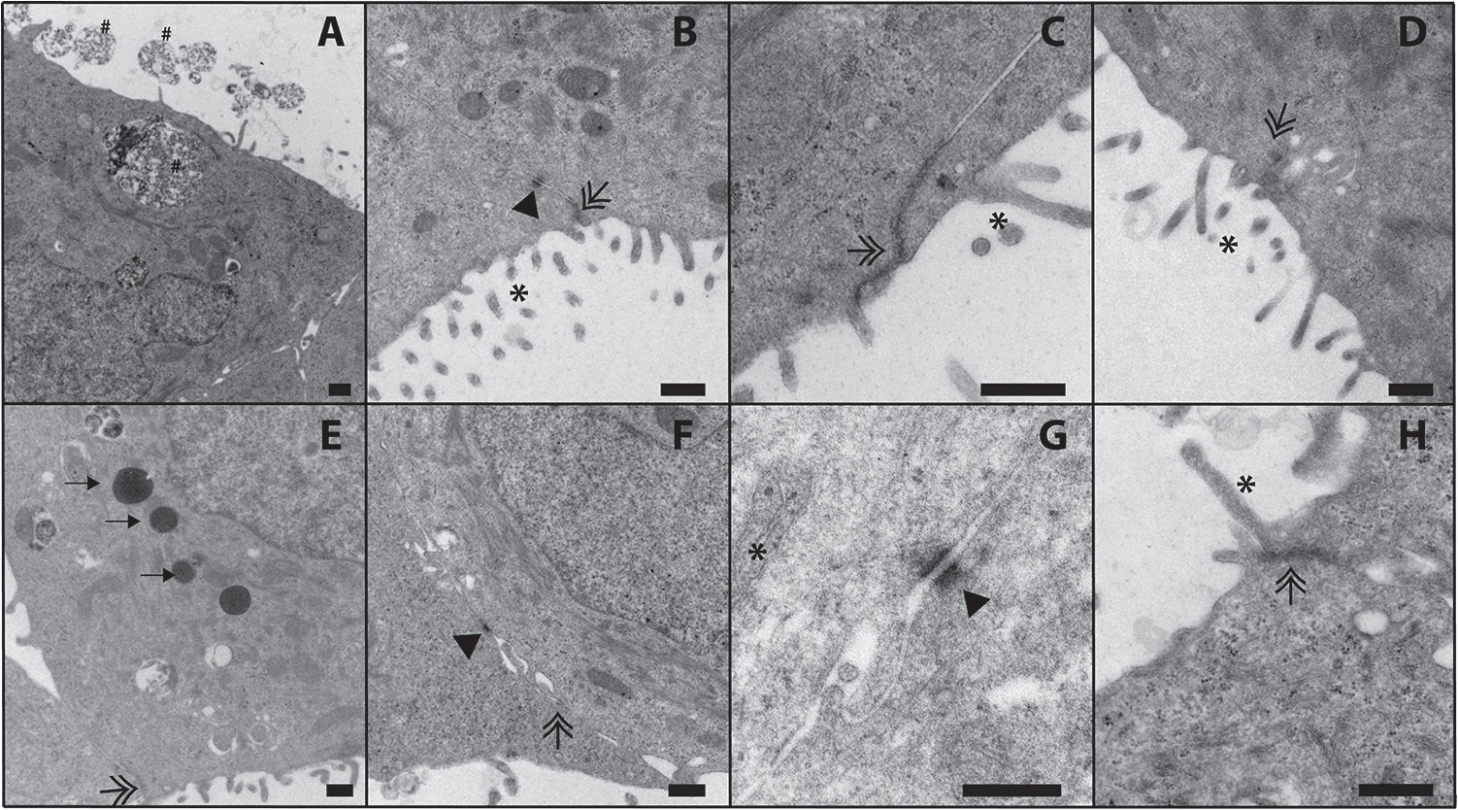
Transmission electron microscopy of 7 day MCF-7 microtissues. Following 7 days of growth in non-adhesive agarose hydrogels, 3D MCF-7 microtissues exhibit luminal secretions (A, hastags), microvilli on the apical surface (B-D, asterisks), tight junctions (B-F, G, double arrows), desmosomes (B, F, G, arrowhead) and lipid droplets (E, arrows). Scale bar = 500nm.

TEM was used to assess the ultrastructural morphology of MCF-7 microtissues and the PAS-positive secretions in the luminal spaces. MCF-7 cells within microtissues possessed intracellular secretory vesicles that were released into the luminal space (Fig 6A). Microtissues had microvilli on the interior apical surface, along with tight junctions present at the apical surface (Fig 6B, 6C, 6D, 6F and 6H). MCF-7 microtissues also contained lipid droplets (Fig 6E) and desmosomal junctions (Fig 6F and 6G).

**Fig 7:**
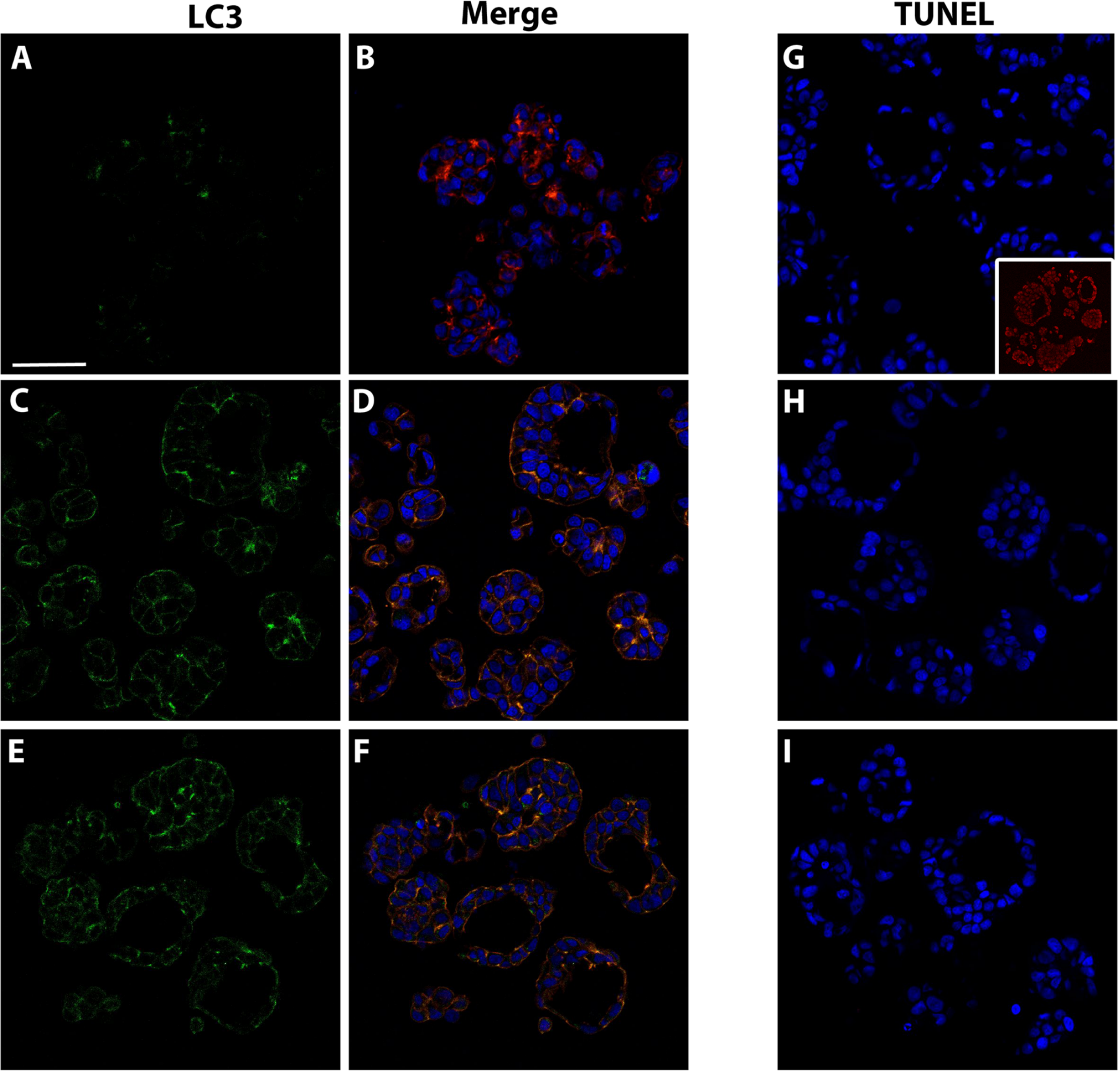
Lumen formation in MCF-7 microtissues is not a result of apoptosis or autophagy. Following 3 days in culture (A, B), LC3 staining is diffuse within cells. LC3 staining (green) increases over time in culture at 5 (C, D) and 7 (E, F) days, but is not localized to vacuoles. MCF-7 cells cultured for 3(G), 5 (H) and 7(I) days do not possess any TUNEL positive nuclei (red) when compared to DNAse-treated positive control (D, inset). Scale bar = 80μm.

Staining for autophagy marker LC3 and TUNEL were used to determine the role of cell death in lumen formation. Following 3 days in culture (Fig 7A), MCF-7 microtissues demonstrated diffuse staining for LC3. Over time in culture, MCF-7 cells have increased, diffuse staining for LC3 at 5 (Fig 7B) and 7 days of culture (Fig 7C). Compared to the DNAse-treated positive control (Fig 7A, inset) TUNEL staining revealed no TUNEL-positive nuclei or debris (red) at 3, 5, or 7 days in culture (Fig 7D–7F).

**Fig 8:**
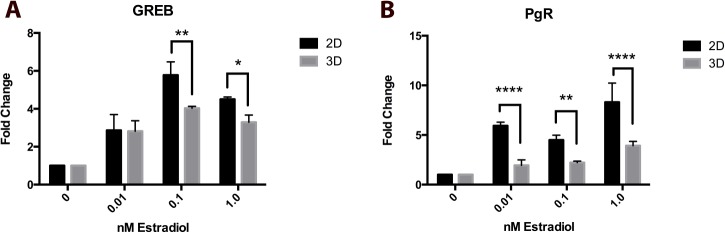
Estradiol responsiveness of MCF-7 2D and 3D cultures. 2D and 3D samples are responsive to 17β-estradiol in a concentration-dependent manner, as evidenced by increased expression of GREB (A) and PGR (B) following 8 hours of exposure. MCF-7 monolayer and hydrogel culture respond to estradiol, with monolayer cultures responding more robustly. * = p≤0.05, ** = p≤0.01 p*** = p≤0.001, **** = p≤0.0001.

To determine 3D MCF-7 microtissue responsiveness to estrogen compared to 2D cultures, 2D and 3D samples were treated with 0.01, 0.1 and 1nM concentrations of 17β-estradiol for 8 hours (Fig 8). Estrogen responsiveness was assessed by RT-PCR for known targets of estrogen receptor, including estrogen-induced growth, breast cancer (GREB) and progesterone receptor (PGR). Monolayer and microtissue MCF-7 cultures responded in a concentration dependent manner to 0.01, 0.1 and 1nM estradiol as demonstrated by increased GREB (Fig 8A) and PGR (Fig 8B) expression compared to untreated, time-matched control samples. Monolayer MCF-7 cells demonstrated significantly higher expression of both PGR and GREB than 3D cultures following exposure to estradiol, particularly at 0.1 and 1 nM concentrations.

## Discussion

When cultured in a scaffold-free, non-adhesive agarose hydrogel system, MCF-7 breast carcinoma cells derived in our lab self-assemble and organize into microtissue structures with luminal spaces surrounded by 1–2 epithelial cell layers, in contrast with the typical 2D morphology which consists of cells growing in a cobblestone-like pattern. The luminal structure of the MCF-7 microtissues shares features with both the *in vivo* morphology of the normal adult breast epithelium that has been previously described [36] and the mammary acini formed by normal MCF-10A cells grown in Matrigel [15]. As a comparison, MCF-7 cells purchased from the same lot from ATCC and derived using identical protocols were obtained from the Yager lab and cultured in this model. Interestingly, the sublines derived at each location exhibit different morphologies when cultured in scaffold-free hydrogels, indicating that the 3D structures formed in this model are due to properties intrinsic to the cells, and are not a result of the culture system.

In agreement with previous work based on MCF-10A cells grown in Matrigel, MCF-7 cell microtissues demonstrate intense staining for MUC1 and MFGE8 localized within selected cells [15], indicating that MCF-7 cells cultured in this model have functions similar to those seen in non-malignant MCF10A cells. However, within MCF-10A 3D cultures, MUC1 and MFGE8 are located at the apical surface, while MCF-7 cells within this model show localization at the basolateral surface. When compared to the staining profile in 2D cells, MCF-7 cells exhibit stronger, localized staining for both of these markers, correlating with the increased mRNA expression detected in the present study.

Additionally, the staining pattern for GM130 was diffuse in 2D MCF-7 cultures, and in 3D cultures cells displayed punctate, directional staining. When compared to 2D cultures, the staining for these markers is more localized and directional, indicating that 3D culture alters the secretory pathways and allows MCF-7 cells to begin to polarize. GATA3 expression and localization in the nucleus indicates that MCF-7 microtissues are forming luminal-like tissues, as GATA3 has been shown to be a key regulator in luminal fate and differentiation [37]. Coupled with GATA3 expression, MCF-7 cells in 3D stain intensely for luminal cytokeratin 8 [38, 39]. The epithelial phenotype marker CDH1 is more strongly localized to cell-cell contacts and strongly expressed in 3D cultures, compared to the cytoplasmic staining observed in 2D MCF-7 cultures. This is corroborated by the high expression of the mesenchymal marker VIM in 2D cultures. The strong staining for KRT8 is confounded by positive staining for the basal marker KRT5; however, KRT5 staining is decreased and more localized in 3D cultures than in 2D, suggesting that over time in 3D culture, MCF-7 cells are exhibiting a more luminal phenotype.

This finding can also be explained by the heterogeneity observed in MCF-7 cells, such as KRT5-positive MCF-7 sublines that may correspond to therapy-resistant cell types in breast cancers [40]. Based on the staining of these markers, MCF-7 cells cultured in agarose hydrogels for 7 days differentiate and exhibit several characteristics of luminal-type cells when compared to 2D MCF-7 cultures. It is worth noting that the current study examines cultures of different ages, and this may contribute to some of the differences observed in marker expression. However, based on the histology and organization of the 3D microtissues and expression of markers, it can be concluded that the 3D MCF-7 microtissues are more differentiated than 2D cultures.

As expected, MCF-7 cells cultured as monolayers have different gene expression patterns compared to those cultured in 3D. After 7 days in scaffold-free culture, MCF-7 microtissues show increased expression of mammary epithelial associated genes KRT8, KRT19, MFGE8 and MUC1 and decreased expression of VIM, KRT14 and BMP7. The increased expression of KRT8 in concert with decreased expression of KRT14 in 7 day MCF-7 microtissues suggests that the cells are further differentiating into luminal-like epithelial cells when compared to MCF-7 cells grown in 2D monolayer culture, since breast luminal epithelial cells express high levels of KRT8 and KRT19 while basal cells express KRT14 [38, 41]. Increased expression of MUC1 mRNA and protein indicates that cells cultured in 3D are further differentiated and may be increasing their secretory functions[39]. Previous work examining mammary gland remodeling has indicated that MFGE8, a milk glycoprotein, is highly expressed in the mammary epithelium, and plays an essential role in mammary gland involution, and the increased expression of this mRNA and protein in 3D MCF-7 microtissues signals that within this model, cells are further differentiating and undergoing a unique morphogenic process that cannot be modeled in 2D systems [42]. The increased expression of MUC1 and MFGE8 proteins coupled with the basolateral, directional staining profiles during culture in 3D implies that cells within this model have secretory function, in contrast to monolayer cultures that display diffuse GM130 and MFGE8 staining. Combined with the presence of secretory vesicles evident by both PASH staining and TEM, this data demonstrates that MCF-7 microtissues are more differentiated in 3D scaffold-free culture than in 2D culture.

The development and remodeling of the mammary epithelia is a dynamic process dependent on many molecular pathways. Coupled with the increased expression of luminal markers is the decreased expression of APOD. APOD is involved in lipid transport, specifically cholesterol transport, and is decreased in 7day 3D cultures. In a recent study of differentiating HC11 mammary epithelial cells, alterations in phospholipid biosynthesis were observed, demonstrated by a decrease in many markers of cholesterol transport including APOD during mammary differentiation [43].

Also indicating that MCF-7 3D cultures are more differentiated than 2D monolayer cultures is the change in expression of several markers of cell invasion and growth, including SNAI1, VIM, BMP7, CCND2, and WISP2. Compared to 2D cultures, the transcriptional regulator SNAI1 is highly expressed, seemingly confounding the observation above that cells are more differentiated as demonstrated by increased KRT8 and KRT19 mRNA expression, as increased SNAI1 expression typically correlates with increased invasive potential [44]. Increased SNAI1 mRNA expression observed in this study counters the decreased expression of the mesenchymal marker VIM, and the non-significant changes in CDH1 expression. Additionally, despite increased SNAI1 mRNA, there is no significant increase in SNAI1 protein in 3D MCF-7 cultures. We hypothesize that this signaling pathway may be dysregulated in these cells, and this warrants further investigation. However, based on recent studies, it is possible that the increase in SNAI1 mRNA expression observed in 3D MCF-7 microtissues may be linked to the morphogenic changes observed over time in these cultures. Previous work has implicated SNAI1 and other Snail family members in mammary morphogenesis, specifically in the process of tubulogenesis [45]. SNAI1 has also been shown to directly regulate CYP19A1, repressing its expression [46]. Increased CYP19A1 expression has been correlated with poor prognosis in breast cancer [47], and the decreased expression observed in the current study further supports that MCF-7 cells are becoming more differentiated when cultured in scaffold-free conditions. Mesenchymal marker VIM is also down-regulated in 3D microtissues when compared to cells cultured in 2D, indicating that MCF-7 cells cultured in 3D are more epithelial-like. In this model, MCF-7 cells decrease expression of the cell cycle regulator CCND2, indicating that cells are changing their rate of cell division, compared to cells in 2D [48], corroborating previous work using MCF-7 cells in a scaffold-based 3D model [49]. Additionally, increased expression of WISP2 in 7 day 3D cultures indicates that the cultures are altering their growth patterns, perhaps by slowing cell cycle progression [50]. Previous research has demonstrated that a loss of WISP2 expression promotes epithelial-to-mesenchymal transition in MCF-7 breast cancer cells, leading to invasive growth, while overexpression in invasive MDA-MB-231 cells inhibits proliferation and reduces cell migration [51]. The increased expression of WISP2 in 3D culture coupled with the altered gene expression of classical cell cycle regulator CCND2, further supports the notion that MCF-7 microtissues do not become more invasive in this model, and instead are slowing proliferation and becoming more epithelial-like, similar to previous work in 3D models [50]. The significantly decreased expression of BMP7 supports this interpretation, as BMP7 expression has been correlated with mesenchymal-to-epithelial transition and increased cell growth [52]. It appears that despite being cancer cells, MCF-7 microtissues slow their proliferation to reach a more quiescent state, in agreement with previous work in normal breast cells cultured in 3D in basement membrane [53] when compared to 2D monolayer culture.

MCF-7 microtissues contained PAS positive secretions in their luminal spaces, suggesting that these cells had secretory function following 7 days in culture. The quantity of PAS positive foci in the luminal space increased as culture time increased, and the preferential secretion into the luminal space demonstrated that MCF-7 cells within this model were organized with distinct directionality, a feature strongly lacking in most monolayer cultures. To better characterize the polarization of MCF-7 cells cultured within this model, microtissues were analyzed using TEM. The presence of microvilli on the interior, luminal cell surface in 7 day MCF-7 microtissues indicates that the cells were undergoing polarization, with the apical surface facing the luminal space and the basal-like surface on the exterior of the microtissue. As revealed by TEM, MCF-7 cells also contained membrane-bound secretions in the luminal space. Madin-Darby canine kidney (MDCK) epithelial cells grown in 3D are polarized, and have been used to demonstrate the important role of cell polarity on formation of functional epithelial barriers, proper cellular transport [54] and cell-type specific functions [13, 55]. This previous work has also demonstrated that loss of cell polarity can lead to disease pathogenesis, calling into question the use of non-polarized 2D cell culture methods to study disease states, and lending credibility to the use of polarized 3D models to study both disease progression and potential therapeutics. Unlike MCF-10A cells, MCF-7 cells within this model appear to be hemi-polarized, with microvilli and PAS-positive secretions present on the interior, apical surface. GM130, MFGE8 and MUC1 staining appear to reveal inverted polarity of MCF-7 microtissues compared to MCF-10A acini in Matrigel, however, this is not surprising due to the cancerous origins of the MCF-7 cell line. The ability of MCF-7 cells to polarize in this model without added extracellular matrix is an important finding, as the use of a scaffold-free culture system eliminates the difficulty of choosing an appropriate matrix material. Previous studies have demonstrated that MCF-7 cells cultured on collagen I have reduced viability compared to those cultured on reconstituted basement membrane [56], indicating that the selection of matrix is of importance and can influence downstream results including gene expression [57]. The use of a scaffold-free culture model allows the focus to be placed on cell-cell interactions, in contrast to focus on cell-matrix interactions in many 3D models.

Interestingly, MCF-7 microtissues displayed no apoptotic cells in the luminal space, in contrast to previous work using the nonmalignant human breast cell line MCF-10A. When grown in 3D in Matrigel, MCF-10A cells organize into cellular aggregates and then undergo apoptosis to form acini containing luminal spaces [15, 58]. The lack of TUNEL-positive nuclei and apoptotic debris in the lumens of MCF-7 microtissues suggests that the luminal spaces may form in a process similar to cyst formation seen with MDCK cells cultured in 3D with Matrigel [59]. The closer resemblance of MCF-7 microtissues to 3D MDCK cultures than MCF-10A cultures may be due to the lack of caspase-3 expression in MCF-7 cells [60]. In the present study, pro-apoptotic transcripts BAD, BAX and CASP3 and anti-apoptotic transcripts BCL2 and BCL2L2 were not significantly changed between 2D and 3D samples, further indicating that MCF-7 microtissues do not undergo lumen formation in an apoptosis-dependent manner. Staining for the autophagy marker LC3 increased over time from days 3 to 7 in culture, but this staining was diffuse throughout the cells, and did not stain any material in the luminal spaces of microtissues. In contrast to previous work using MCF-7 cells to form spheroids in non-adherent plates [61], MCF-7 microtissues in the present study did not display large autophagic vacuoles or autolysomsomes as detectable by TEM. Past studies have demonstrated that combined inhibition of both autophagy and apoptosis is not sufficient to inhibit luminal clearing in MCF-10A acini [58], indicating that other pathways may play a role in lumen formation in epithelial tissues [62, 63] While this observation warrants further investigation, this system may be useful in studying pathways of lumen formation in the mammary gland.

The use of estrogen receptor positive MCF-7 cells [64] in this model provides a unique 3D system for assessing estrogen signaling and the effects of estrogen on mammary microtissues. To determine if MCF-7 cells cultured in 3D maintain their estrogen responsiveness, microtissues were treated with 17β-estradiol for 8 hours, and gene expression of known estrogen targets was examined. When compared to untreated controls, MCF-7 cells demonstrate a robust response to estrogen stimulation, indicated by significantly increased expression of PGR and GREB. However, 2D cultures exhibited a stronger induction of GREB and PGR, targets of estrogen receptors. This correlates with the observation that within this model, MCF-7 cells cultured in agarose hydrogels significantly decrease expression of ESR2. Despite this, MCF-7 microtissues remain responsive to estrogens. The use of more physiologically relevant MCF-7 3D cultures in place of 2D cultures to assess estrogenic activity of compounds has the potential to provide more complete information for screening purposes, and may allow for the evaluation of morphological endpoints in addition to molecular changes.

The current study presents the first characterization of MCF-7 microtissues grown in 3D using non-adhesive agarose hydrogels. Microtissues formed in this model are more differentiated than 2D cultures as demonstrated by both their structural and molecular profiles. The more differentiated state of MCF7 cells grown as 3D microtissues allows for their protracted use as breast cell models in the investigation of estrogen receptor biology, therapeutics, and the toxicological effects of estrogens and xenoestrogens. The formation of differentiated structures free of the influence of external matrices demonstrates the utility and simplicity of this system, a system amenable to high-throughput analysis.

## Supporting Information

S1 Fig MCF-7 cells derived in separate locations using identical culture methods exhibit different morphologies in 3D. MCF-7 cells derived in another lab were cultured for 5 (A), 7 (B) and 10 (C) days in 3D. MCF-7 cells were purchased from the same lot number from ATCC and derived in two locations utilizing identical protocols. When cultured in scaffold-free agarose hydrogels, MCF-7 cells derived by the Yager lab form large microtissues that do not contain defined luminal spaces up to 10 days in culture.
(TIF)

S1 Table Antibodies used for immunofluorescence. Antibody catalog numbers and dilutions used for immunofluorescence on 3D MCF-7 cryosections.
(PDF)

S2 Table Antibodies used for Western blotting. Antibody catalog numbers and dilutions used for Western blotting.
(PDF)

S3 Table List of genes selected for the custom SABiosciences PCR Array. Gene names, abbreviations and main functions of gene included on the custom PCR arrays.
(XLS)

S4 Table Complete PCR-results for PCR arrays. Fold change values of all RT-PCR targets, sorted by ascending q-value.
(XLSX)
