# Supplementary figures and images for: MCF-7 Human Breast Cancer Cells Form Differentiated Microtissues in Scaffold-Free Hydrogels
Source: PLoS One. 2015 Aug 12;10(8):e0135426. doi: 10.1371/journal.pone.0135426 (PMC4534042; doi:10.1371/journal.pone.0135426)

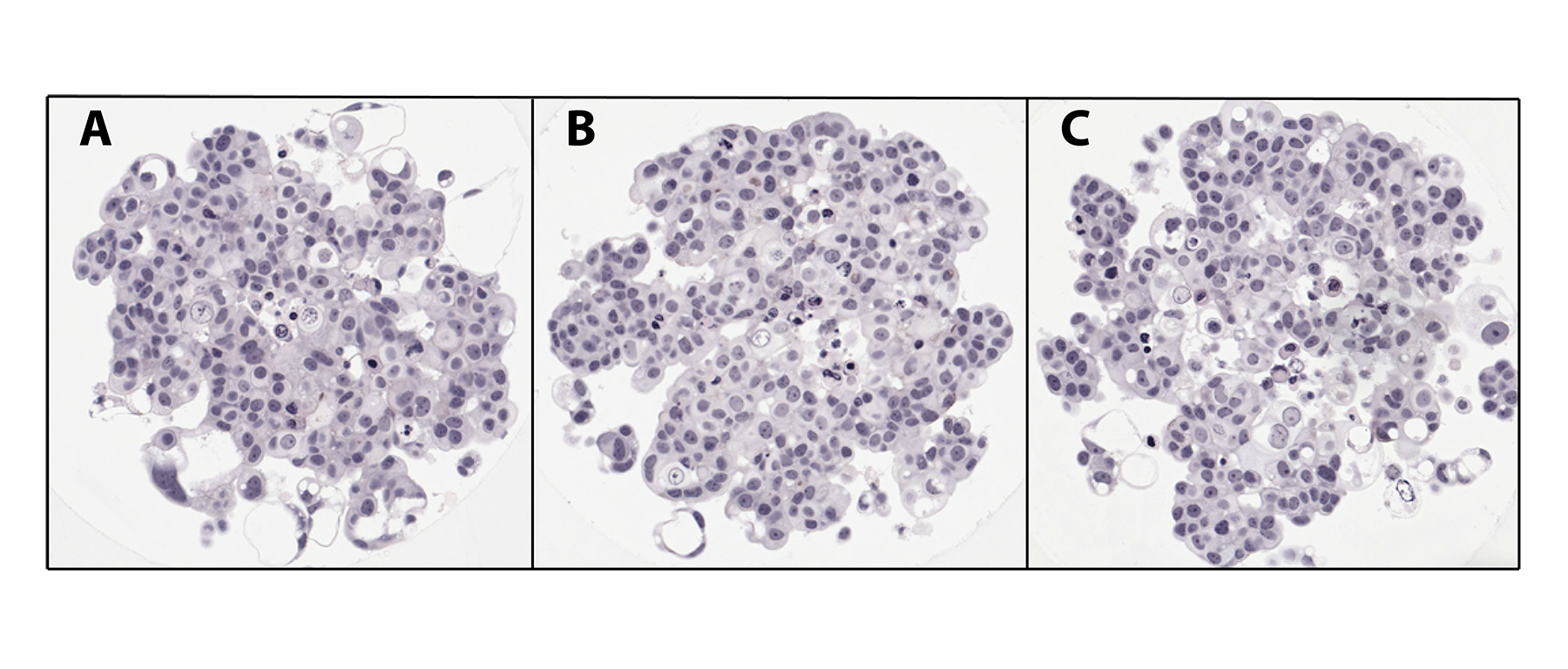

Supplement: S1 Fig — MCF-7 cells derived in another lab were cultured for 5 (A), 7 (B) and 10 (C) days in 3D. MCF-7 cells were purchased from the same lot number from ATCC and derived in two locations utilizing identical protocols. When cultured in scaffold-free agarose hydrogels, MCF-7 cells derived by the Yager lab form large microtissues that do not contain defined luminal spaces up to 10 days in culture. (TIF) [file pone.0135426.s001.tif]
