# Supplementary material for: MCF-7 Human Breast Cancer Cells Form Differentiated Microtissues in Scaffold-Free Hydrogels
Source: PLoS One. 2015 Aug 12;10(8):e0135426. doi: 10.1371/journal.pone.0135426 (PMC4534042; doi:10.1371/journal.pone.0135426)
Supplement: S1 Table — Antibody catalog numbers and dilutions used for immunofluorescence on 3D MCF-7 cryosections. (PDF) [file pone.0135426.s002.pdf]

Protein	Abbreviation	Primary Antibody	Primary Dilution	Secondary Antibody	Secondary Dilution
Milk fat globule-EGF factor 8	MFGE8	Abcam #ab17787	1:2000	AlexaFluor #A11001	1:500
Mucin 1	MUC1	Abcam #ab15481	1:1000	AlexaFluor #A11008	1:500
Trans-acting T-cell-specific transcription factor GATA-3	GATA3	Cell Signaling #5852	1:500	AlexaFluor #A11008	1:500
Keratin 8	KRT8	Abcam #ab9023	1:100	AlexaFluor #A11001	1:500
Keratin 5	KRT5	Abcam #ab24647	1:100	AlexaFluor #A11008	1:500
E-cadherin	CDH1	BD #610181	1:500	AlexaFluor #A11001	1:500
GM130	GM130	Cell Signaling #12480	1:500	AlexaFluor #A11008	1:500
Human Disc Large	hDlg	Santa Cruz Sc-9961	1:200	AlexaFluor #A11001	1:500
Vimentin	VIM	Sigma Aldrich V6630	1:200	AlexaFluor #A11008	1:500
