# Supplementary material for: MCF-7 Human Breast Cancer Cells Form Differentiated Microtissues in Scaffold-Free Hydrogels
Source: PLoS One. 2015 Aug 12;10(8):e0135426. doi: 10.1371/journal.pone.0135426 (PMC4534042; doi:10.1371/journal.pone.0135426)
Supplement: S2 Table — Antibody catalog numbers and dilutions used for Western blotting. (PDF) [file pone.0135426.s003.pdf]

Protein	Abbreviation	Primary Antibody	Primary Dilution	Secondary Antibody	Secondary Dilution
Snail family zinc finger 1	SNAI1	Cell Signaling #3879	1:500	Cell Signaling #7074	1:1000
Wnt-induced signaling protein 2	WISP2	Abcam #ab38317	1:500	Cell Signaling #7076	1:1000
Milk fat globule-EGF factor 8	MFGE8	Abcam #ab17787	1:2000	Cell Signaling #7076	1:1000
Mucin 1	MUC1	Abcam #ab15481	1:1000	Cell Signaling #7074	1:1000
Vimentin	VIM	Sigma #V6630	1:1000	Cell Signaling #7076	1:1000
Amphiregulin	AREG	Abcam #ab180722	1:1000	Cell Signaling #7074	1:1000
Glyceraldehyde 3-phosphate dehydrogenase	GAPDH	Cell Signaling #2118	1:5000	Cell Signaling #7074	1:5000
